# Squamous cell carcinoma in mature cystic teratoma of the ovary induced by human papillomavirus 16 infection: A case report and literature review

**DOI:** 10.1097/MD.0000000000030667

**Published:** 2022-09-23

**Authors:** Zhixian Shi, Lingyun Yang, Ce Bian

**Affiliations:** a Department of Gynecology and Obstetrics, Key Laboratory of Birth Defects and Related Diseases of Women and Children, Ministry of Education, West China Second University Hospital, Sichuan University, Chengdu, Sichuan Province, China; b Department of Gynecology and Obstetrics, Key Laboratory of Birth Defects and Related Diseases of Women and Children, Ministry of Education, West China Second University Hospital, Sichuan University, Chengdu, Sichuan Province, China.

**Keywords:** case report, human papillomavirus 16, mature cystic teratoma, ovarian squamous cell carcinoma

## Abstract

Mature cystic teratoma is the most common ovarian germ cell tumor. The malignant transformation of ovarian mature cystic teratoma (MCT) is very rare, but the prognosis is poor. We present a case of ovarian mature cystic teratoma with human papillomavirus infection and malignant transformation into ovarian squamous cell carcinoma (SCC). The occurrence of this case may prove that high-risk human papillomavirus infection is a pathogenic factor inducing malignant transformation of mature cystic teratoma to SCC.

**Patient concerns:**

A 38-year-old woman with a solid cystic mass of 8 cm on the right ovary, and human papillomavirus (HPV) test of her cervix showed HPV-16 infection.

**Diagnosis:**

The transvaginal ultrasound was performed, and there was a cystic solid mass of 5.9 × 4.5 × 5.5 cm in the right adnexal area with unclear cystic fluid and rich blood flow signals in the capsule wall. HPV test of cervix showed HPV-16 infection. Diagnostic suspicion: cystic teratoma.

**Intervention:**

The patient signed an laparoendoscopic surgery was performed to remove the right ovarian mass. Intraoperative pathology consultation revealed the malignant transformation of mature teratoma of the right ovary and the formation of squamous or adeno-SCC. We performed laparoscopic comprehensive surgical staging (hysterectomy, bilateral salpingo-oophorectomy, omentectomy, appendectomy, pelvic and para-aortic lymph node dissection) were made.

**Outcomes:**

The operation was successful and the postoperative recovery was smooth, was discharged 7 days after operation. Now the patient is recovering well and is continuing chemotherapy as planned.

**Conclusion:**

HR-HPV infection might be a causal factor for inducing malignant transformation of ovarian MCT to SCC, and the Jumping metastasis of lymph nodes may be the characteristic of SCC-MCT, but further verification is still needed.

## 1. Introduction

Mature cystic teratoma (MCT), also known as the dermoid cyst, is the most common ovarian germ cell tumor, accounting for 10% to 20% of all ovarian tumors.^[1]^ Histologically, MCT contains 3 germ layers (ectoderm, mesoderm, and endoderm). The malignant transformation of MCT can occur in any of the 3 germ layers, and its malignant transformation rate is low. Among all ovarian MCTs, the incidence of squamous cell carcinoma (SCC) in MCT (SCC-MCT) is 0.17% to 2%, and 80% of SCC-MCT is derived from ectoderm.^[2]^ However, compared with epithelial ovarian cancer, the prognosis of SCC-MCT is significantly worse. The mechanism and relevant biomarkers of SCC-MCT are still unrevealed, because there are few reports discussing about it. Patients with SCC-MCT do not present any typical symptoms and signs in the early stage and are diagnosed unexpectly based on intraoperative or postoperative pathologic examination.

As we know, high-risk human papillomavirus (HR-HPV) infection, especially HPV-16 and HPV-18, is highly associated to many kinds of SCC, such as cervical, vaginal and vulvar, and oropharyngeal SCC, etc. Limited reports considered that HR-HPV is mostly to blame for the primary ovarian SCC.^[3–6]^ However, there was no evidence to prove that MCT-SCC could be induced by HR-HPV infection.

We herein present a case that SCC and HPV-16 were both discovered in ovarian MCT. We considered that the malignant transformation of ovarian MCT, especially SCC, could be induced by HR-HPV from genital tract infection. Consequently, patients with both ovarian MCT and genital tract HR-HPV infections should be vigilant about the malignant transformation of SCC-MCT, and it will provide a relevant preventive measure for SCC-MCT.

## 2. Case report

A 38-year-old woman presented to our hospital with her annul medical examination report which emphasized that there was a solid cystic mass of 8 cm on the right ovary. Upon obtaining a complete clinical history, the patients reported that she had no lower abdominal pain, and the menstrual cycle was regular (29–30 days; 6–7 days duration) with no cessation of menstruation or irregular bleeding. The transvaginal ultrasound was performed, and there was a cystic solid mass of 5.9 × 4.5 × 5.5 cm in the right adnexal area with unclear cystic fluid and rich blood flow signals in the capsule wall (diagnostic suspicion: cystic teratoma). Interestingly, no abnormal tumor markers were found (CA125 19.3 U/mL; CA199 21.4 U/mL; CA153 13.9 U/mL; CEA 0.5 ng/mL; HCG < 2.0 mIU/mL). Her medical examination report showed that the Pap smear screening was negative this year; however, HPV test of her cervix showed HPV-16 infection. The patient signed an informed consent and laparoendoscopic surgery was performed to remove the right ovarian mass. We found that the right ovary is enlarged, with a 6 × 5 × 4 cm solid cystic mass containing the lipid and scalps tissues. Interestingly, the presence of scattered dark brown nodules inside the cyst was found. The endocystic wall is thin and smooth. We removed the right ovarian mass completely during operation. Intraoperative pathology consultation revealed the malignant transformation of mature teratoma of the right ovary and the formation of squamous or adenoSCC. Tumor stage was preliminary determined as stage 1A according the FIGO stage. Due to no fertility desired for this patient, after getting the verbal consent from her husband, we performed laparoscopic comprehensive surgical staging (hysterectomy, bilateral salpingo-oophorectomy, omentectomy, appendectomy, pelvic and para-aortic lymph node dissection) were made.

On entering the abdomen, peritoneal lavage were collected for peritoneal cytologic test. And then, describing a normal uterus, left ovary presented with normal appearance, with adhesions to epiploic appendages from the tube. The lymph nodes around the abdominal aorta and the surface of the inferior vena cava were typically enlarged with dark brown pigmentation, however, no enlarged pelvic lymph nodes were found. Intestine, omentum, appendix, peritoneum, small intestine, diaphragmatic domes, liver, and stomach without implants. Intraoperative pathology consultation revealed the lymph nodes around the abdominal aorta was infiltrated by the tumor. Para-aortic lymph node dissection was performed by stripping the nodal tissue from the vena cava and the aorta bilaterally to the level of the renal vessels. The method of dissecting pelvic lymph nodes is bilateral removal of lymph nodes overlying and anterolateral to the common iliac vessel, overlying and medial to the external iliac vessel, overlying and medial to the hypogastric vessels, and from the obturator fossa at a minimum anterior to the obturator nerve.

**Figure 1.**
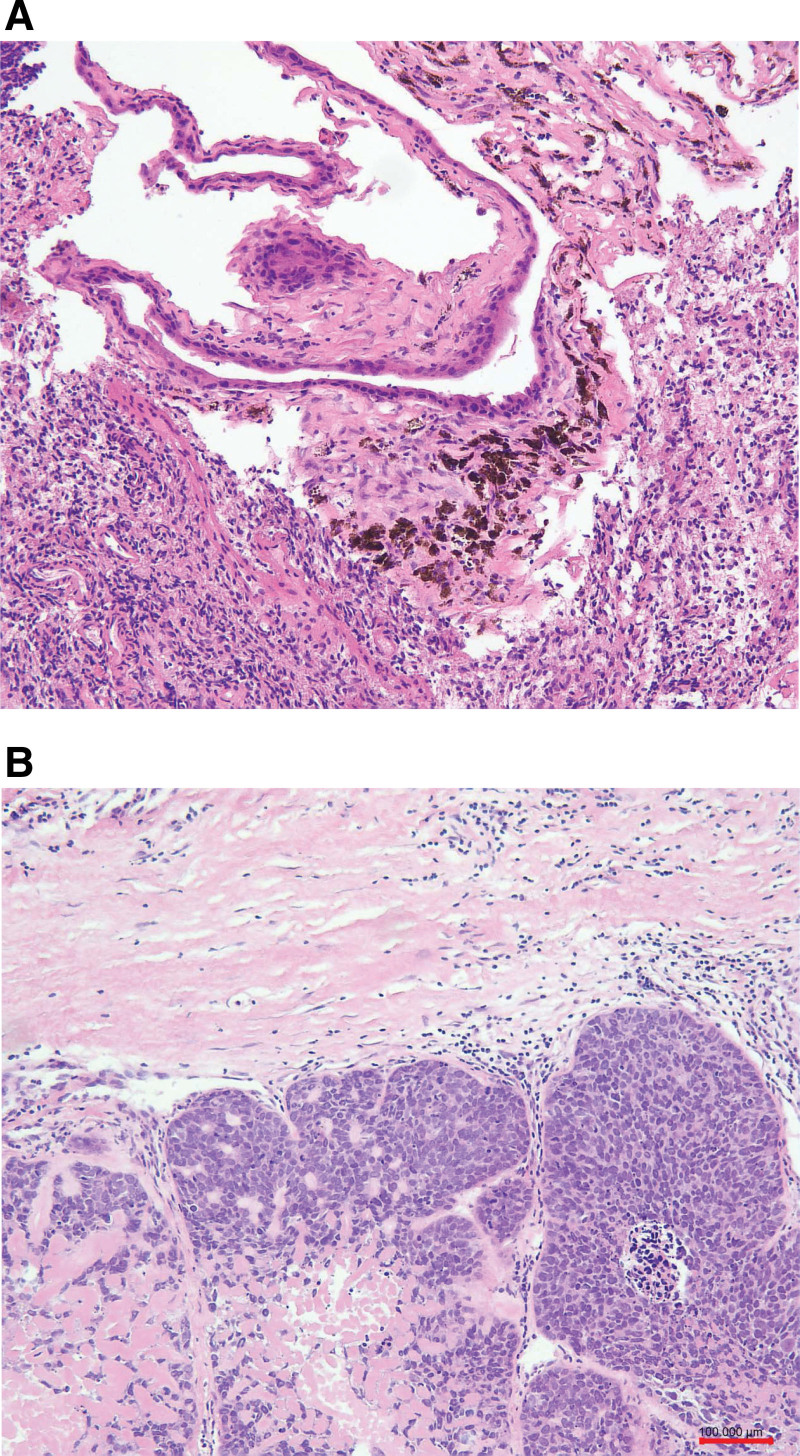
(A) The benign elements in the MCT (H&E, ×10 obj). (B) SCC transformed from the MCT (H&E, ×10 obj) with hyperchromatic nuclei. H&E = hematoxylin and eosin, MCT = mature cystic teratoma, SCC = squamous cell carcinoma.

**Figure 2.**
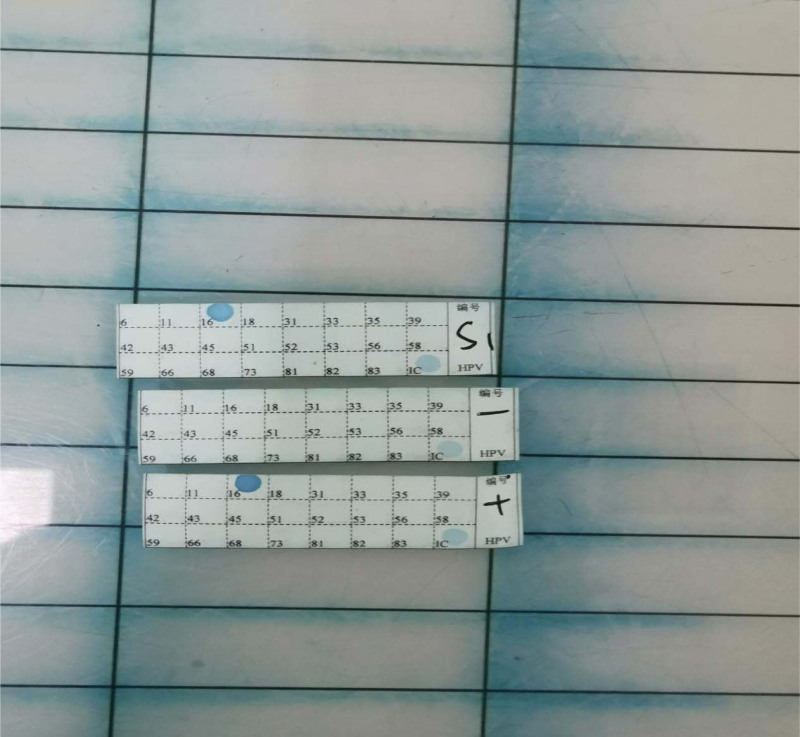
The PCR-RDB was utilized to detect the HPV subtypes [Yaneng BIOscience (shenzhen) Co. Ltd.]. HPV = human papillomavirus, PCR-RDB = PCR reverse dot blot hybridization technique.

A final diagnosis of malignant transformation of mature teratoma of the right ovary, which was divided into medium to poorly differentiated SCC (Fig. 1). And HR-HPV-16 was found in the right ovary (Fig. 2). The lymph nodes around the abdominal aorta were infiltrated by the tumor. Uterus, left ovary, omentum, appendix, and the pelvic lymph nodes were free of tumor deposits. Ovarian surface was free of tumor and lymphovascular invasion was not identified. Consequently, tumor stage was determined as stage IIIC according the FIGO stage.

The operation was successful and the postoperative recovery was smooth, was discharged 7 days after operation. Now the patient is recovering well and is continuing chemotherapy as planned.

## 3. Patient consent

Informed consent was obtained from the patient for the publication of case details and images.

## 4. Discussion

The incidence rate of ovarian SCC is low, most of which originate from malignant transformation of teratoma, Brenner tumor, or endometriosis.^[7]^ Teratoma is a germ cell tumor that may consist of mature or immature tissues derived from 3 germ cell layers. MCT accounts for 62% of all ovarian tumors in women under the age of 40.^[8]^ The treatment mainly depends on surgical resection. Its malignant transformation rate is low. Among all ovarian MCTs, the incidence of malignant transformation to SCC is 0.17% to 2%, but its prognosis is very poor. However, there are few cases of HPV in ovarian SCC. To our knowledge, only 4 cases of primary ovarian SCC associated with HPV have been reported so far.^[3–6]^ For the malignant transformation of MCT to SCC with HPV infection, there has been no relevant case report. Due to its low incidence rate, few related studies, and unclear pathogenesis, its diagnosis mainly depends on postoperative examination, which makes its prevention, diagnosis, and treatment face great challenges. A multicenter retrospective study in Taiwan^[9]^ suggested that patients with tumors that spread beyond the ovary (FIGO stages II–IV) had a very low probability of long-term survival. The 5-year survival rate for stage I patients was 93%, indicating that the early detection of malignant transformation before the occurrence of invasion or metastasis is essential for treating SCC. However, at present, the mechanism of MCT to SCC is not completely clear, which makes its early treatment and early detection a big problem. Therefore, it is particularly important to explore the mechanism of malignant transformation from MCT to SCC and detect its malignant transformation potential early in clinical practice.

Human papillomavirus (HPV) infection is considered to be the main cause of infection-related cervical cancer, vulvar cancer, vaginal cancer, anal cancer, oropharyngeal cancer, and penile cancer.^[10]^ At present, more than 100 kinds of HPV have been identified, of which at least 20 are related to cervical cancer.^[11]^ HPV infection is usually a self-limiting process, but almost all cervical cancers are associated with HR-HPV infection, suggesting that persistent HPV infection may be an important factor in inducing cancer.^[12]^ Cervical cancer mostly originates from the cervical transformation zone where the columnar epithelium of the cervix changes to the squamous epithelium of the cervix. HPV infects the basal cells of the squamous epithelium of the cervix. Once inside the host cell, the HPV DNA replicates as the basal cells differentiate and progress to the surface of the epithelium. The HPV gene expression becomes unlinked to the state of cellular differentiation of infected epithelial cells, and deregulation expression of the early region of the viral genome results in a dramatic increase in the expression of the 2 HPV oncoproteins (E6 and E7). This results in the loss of the normal cell cycle control of the epithelium, thus leading to the occurrence of diseases.^[13]^ Microscopically, the MCT is usually composed of elements from all 3 germ cell layers: ectoderm (i.e., squamous epithelium, skin/adnexal structures, brain, peripheral nervous system tissue, cerebellum, and choroid plexus), mesoderm (i.e., fat, bone, cartilage, teeth, blood vessels, smooth muscle, lymphoid tissue, skeletal muscle), and endoderm (i.e., respiratory and gastrointestinal epithelium, thyroid, and salivary gland tissue), although often ectodermal derivatives predominate.^[14]^ Therefore, we speculate that HPV infection may act on the squamous epithelium of MCT ectoderm, leading to the malignant transformation of MCT. Chiang et al^[15]^ detected HPV infection in 4 SCC-MCT ovarian samples through IHC and ISH (4/4), suggesting that HPV infection may be related to SCC-MCT, and HPV infection was also detected in the adjacent reproductive tissues of SCC-MCT cases, which indicates that virus particles may spread in the reproductive system, and HPV particles in the cervix and cervical tube may spread to the ovary through the ascending route of the endometrium and fallopian tube. High-risk HPV infection might be a causal factor for inducing malignant transformation of ovarian MCT to SCC. This is consistent with our case. The patient had cervical HPV-16 infection. This time, ovarian teratoma was found, and malignant transformation into SCC. HR-HPV-16 was also found on the ovary. HPV particles in the cervix and cervical tube may spread to the ovary through the ascending route of the endometrium and fallopian tube. It causes HPV virus to act on the squamous epithelium of MCT ectoderm, thus promoting the malignant transformation of MCT. Therefore, we highly suspect that the malignant transformation of ovarian teratoma into SCC may be caused by the infection of HPV-16 in the squamous epithelial cells of teratoma. But it still needs further verification. Therefore, patients with teratoma should pay attention to screening HPV and be alert to the malignant transformation of squamous epithelium into SCC in teratoma caused by HPV infection.

Interestingly, this case of SCC was accidentally found in MCT, however the lymph nodes around the abdominal aorta and the surface of the inferior vena cava were typically enlarged with dark brown pigmentation, no enlarged pelvic lymph nodes were found. And intraoperative pathology consultation revealed the lymph nodes around the abdominal aorta was infiltrated by the tumor. Ovarian cancer is more likely to metastasize via intraperitoneal dissemination than hematogenously or via lymphatics. However, periaortic lymph node metastasis of ovarian cancer occurred at an early stage in our case. It indicates that ovarian teratoma SCC may have periaortic lymph node metastasis at an early stage. A systematic review of 14 articles showed that the mean incidence of lymph node metastases in clinical stages I-II early ovarian cancer was 14.2% (range 6.1–29.6%), of which 7.1% only in the para-aortic region, and 4.3% both in the para-aortic and pelvic region.^[16]^ This may be due to the unique lymph node metastasis pathway of ovarian cancer. In ovarian cancer there are 3 main pathways in lymphatic drainage: along the ovarian vessels to the para-aortic nodes, the round ligament of the uterus to the groin area and the uterine vessels to the iliac lymph compartments.^[17]^ Lymph node metastasis of ovarian cancer often metastasizes with ovarian arteriovenous reflux, especially venous reflux. The vein of the left ovary flows back to the left renal vein and the right to the inferior vena cava. Therefore, the first stop of lymph node metastasis pathway of ovarian cancer is the location of para-aortic lymph node (above the inferior mesenteric artery to the left renal vein). A prospective study^[18]^ by Ditto A et al pointed out that among early epithelial ovarian cancer with lymph node metastasis, the proportion involving lymph node metastasis in the para-aortic region was as high as 86.6%. This is consistent with the unique metastasis pattern of ovarian cancer. However, there is no relevant report about SCC-MCT. This case is an early case, but it is found that the abdominal para-aortic lymph nodes are positive and the pelvic lymph nodes are negative, indicating that the SCC-MCT may have the metastasis of the abdominal para-aortic lymph nodes in the early stage, and the cancer cells may mainly flow back to the renal vein or the inferior vena cava region through the ovarian vein, resulting in the metastasis of the abdominal para-aortic lymph nodes, while the pelvic lymph nodes are negative. This jumping metastasis may be the characteristic of SCC-MCT, but further verification is still needed. Therefore, we should pay attention to identifying the risk of early para-aortic lymph node metastasis for patients with ovarian mature teratoma SCC.

## Acknowledgments

We would like to thank the patient for agreeing to reveal the case details for publication.

## Author contributions

Zhixian Shi took the lead in drafting the article, Lingyun Yang and Ce Bian provided supervision revised article, and finally approved the version to be published. All authors read and approved the final < article.

Conceptualization: Zhixian Shi, Lingyun Yang, Ce Bian.

Data curation: Zhixian Shi, Lingyun Yang.

Formal analysis: Lingyun Yang, Ce Bian.

Investigation: Zhixian Shi.

Methodology: Lingyun Yang.

Supervision: Lingyun Yang, Ce Bian.

Writing—original draft: Zhixian Shi.

Writing—review and editing: Lingyun Yang.
